# Anti-Resonant Hollow Core Fibers with Modified Shape of the Core for the Better Optical Performance in the Visible Spectral Region—A Numerical Study

**DOI:** 10.3390/polym10080899

**Published:** 2018-08-10

**Authors:** Hanna Izabela Stawska, Maciej Andrzej Popenda, Elżbieta Bereś-Pawlik

**Affiliations:** Department of Telecommunications and Teleinformatics, Wroclaw University of Science and Technology, 50-370 Wroclaw, Poland; maciej.popenda@pwr.edu.pl (M.A.P.); elzbieta.pawlik@pwr.edu.pl (E.B.-P.)

**Keywords:** photonic crystal fibers, hollow core fibers, inhibited coupling fibers, negative curvature hollow core fibers, anti-resonant fibers, optical fiber design

## Abstract

In this paper, we present numerical studies of several different structures of anti-resonant, hollow core optical fibers. The cladding of these fibers is based on the Kagomé lattice concept, with some of the core-surrounding lattice cells removed. This modification, by creating additional, glass-free regions around the core, results in a significant improvement of some important optical fiber parameters, such as confinement loss (*CL*), bending loss (*BL*), and dispersion parameter (*D*). According to the conducted simulations (with fused silica glass being the structure’s material), *CL* were reduced from ~0.36 dB/m to ~0.16 dB/m (at 760 nm wavelength) in case of the structure with removed cells, and did not exceed the value of 1 dB/m across the 700–850 nm wavelength range. Additionally, proposed structure exhibits a remarkably low value of *D*—from 1.5 to 2.5 ps/(nm × km) at the 700–800 nm wavelength range, while the *BL* were estimated to be below 0.25 dB/m for bending radius of ~1.5 cm. *CL* and *D* were simulated, additionally, for structures made of acrylic glass polymethylmethacrylate, (PMMA), with similarly good results—*D_PMMA_* ∊ [2, 4] ps/(nm × km) and *CL_PMMA_* ≈ 0.13 dB/m (down from 0.41 dB/m), for the same spectral regions (700–800 nm bandwidth for *D*, and 760 nm wavelength for *CL*).

## 1. Introduction

The appearance of hollow core optical fibers [1] was a true revolution in the field of fiber optics. They are capable of guiding light with remarkably low levels of optical losses and non-linearities, as well as providing the possibility for engineering their dispersion, bi-refringence, etc. [2,3,4,5,6]. Initially, hollow core fibers utilized the photonic bandgap mechanism (through the periodic structure of their cladding) to trap the light within their core, and such fibers are called hollow core photonic bandgap fibers (HC-PBFs). This approach, however, limits the fiber’s transmission bandwidth to certain wavelengths, and the continued search for an alternative mechanism, which could circumvent this problem, resulted in the appearance of hollow core fibers with a microstructured cladding in the form of a Kagomé lattice [7]. Unlike HC-PBFs, the cladding of these fibers does not support photonic bandgaps, and the discussion on the mechanism of their guidance is still ongoing. However, according to the various review papers [8,9,10], the anti-resonant reflective optical waveguide (ARROW) model is currently the most widely accepted one, hence their name—hollow-core anti-resonant fibers (HC-ARFs). The condition of anti-resonance, which is necessary for the light guidance, takes place for the wavelengths that fulfil the following equation [11]:

(1) $$\lambda_{k}=4d_{0}\frac{\sqrt{n^{2}-1}}{2\left(k+1\right)},$$

where *λ_k_* is the wavelength of the considered transmission window (the anti-resonant wavelength), *d*_0_ is the thickness of the first layer of high-index material, surrounding the core (usually silica glass), *k* is 0,1,2, …, defining the transmission’s window order, and *n* is the material’s refractive index. In 2010 and 2011, Wang et al. [12,13] have presented two works which were crucial for the further development of the HC-ARF concept. Firstly, a hypocycloid (negative-curvature) core shape was proposed, allowing for reduction of the confinement loss (*CL*) of HC-ARFs. Secondly, the modal properties of these fibers were proven to be dependent mainly on the geometry of the very first layer of high refractive index material, surrounding the core. It was not very long until a truly single layer, microstructured cladding, hollow-core optical fiber was presented [14]. This idea, due to the simplicity of the cladding structure, and hence its susceptibility to modifications, has been extensively studied, resulting in the appearance of many different types of single-cladding-layer HC-ARFs with negative curvature of the core (we will refer to these fibers as NCHCFs), such as nodeless [15], nested [11,16,17], dual core [18], split-cladding [19], or co-joined tube anti-resonant fibers [20]. All of this interest is well justified when one realizes the potential of the NCHCFs—the overlap of the core mode and cladding area is even lower than in the case of primary (i.e., without the negative-curvature core shape condition) Kagomé fibers, which in turn further reduces any potential absorption and non-linearities of the cladding material, making these fibers ideal candidates for the transmission of ultrashort, high power laser pulses [21,22], as well as making the spectral regions of mid-ultraviolet [23,24] (MUV, 200–300 nm) and near- to mid-infrared (NIR–MIR, 0.8–8 µm) [25,26] available for fiber transmission, without any spectral (due to material absorption) or temporal (due to dispersion) distortions. NCHCFs have also been filled with gases for the purpose of supercontinuum and/or laser light generation [27,28], while recently they have started to appear in the field of biomedical sensing, from multiphoton spectroscopy and microscopy [29,30], to label-free DNA detection [31].

The fact of remarkably low core mode confinement in the NCHCF’s cladding structure has made them a possible enhancement of the field of microstructured plastic optical fibers (m-POF). Indeed, in 2007, Argyros and Pla [32] have presented a polymethylmethacrylate (PMMA) m-POF with a Kagomé lattice, making the transmission in the IR wavelength region (1200–1600 nm) possible with this fiber, which would not be the case for a solid-core POF, as the PMMA material absorption in this spectral region exceeds the value of 10^3^ dB/m [33]. Microstructured, anti-resonant POFs are also of interest for the transmission of THz wavelengths [34,35,36,37], and due to the constant development of 3D-printing technology, structures of increased complexity and reduced size can be expected to become printable in the future. Nevertheless, lots of different mPOFs, fabricated with the conventional extrusion method, have already been presented [38]. It is worth noting that, as stated by [39], plastic optical fibers are already an interesting alternative to their silica-glass counterparts in the field of sensing, due to their increased flexibility, bending resistance and biocompatibility. All of those features, connected with the relatively low cost of POFs, have already made them a well-recognized material for bio-chemical and medical sensing, as well as structural health and environmental monitoring applications [40]. As a result, one can assume that the availability of negative-curvature, hollow-core, anti-resonant POFs, due to their optical properties (high confinement of the light in the hollow core, optical power handling capabilities, remarkably low temporal distortions of the light pulses), can expand the polymer optical fibers potential even more.

In this work, the matter of modifying the structure of the Kagomé hollow core fiber’s cladding, with negative curvature of the core, is further discussed. The concept was stimulated by the previously mentioned papers, as well as our previous work [41], where the improvement of modal properties of the double-clad, hollow-core fiber (DC-HCF), due to the changes of the fiber’s core geometry, was observed. Here, by removing and spreading some of the fiber core-surrounding glass capillaries, it was possible to reduce the *CL* at the wavelength of 760 nm, widely used in multiphoton microscopy, from 0.36 dB/m (unmodified fiber cladding) to ~0.16 dB/m (modified fiber cladding). The bending loss (*BL*) and dispersion parameter (*D*) have also been calculated, suggesting that this type of fiber structure is capable of guiding ultra-short light pulses without any temporal distortions, as well as being weakly susceptible to bending in terms of transmission. The effect of mode-field area reduction for longer wavelengths is also presented. Finally, proposed structures are simulated on PMMA (a standard, polymeric material used for the fabrication of POFs), showing very similar guiding properties (confinement loss spectrum, dispersion parameter) as in the case of their silica-glass counterparts. Due to the fact that a NCHCF with similar structure has already been fabricated [19], we believe that our fiber design is a feasible one. Our goals in this paper were to present that simple modifications of the negative curvature Kagomé HCFs core-cladding interface can significantly influence fiber *CL* and spectral bandwidth, and in turn, we propose a fiber structure that is well-suited for the delivery of high energy, ultrashort laser pulses with a wavelength range of 700 to 800 nm.

## 2. Modified Kagomé Hollow Core Fiber—Modeling and Optical Properties

### 2.1. Modeling of the Structure 

**Figure 1:**
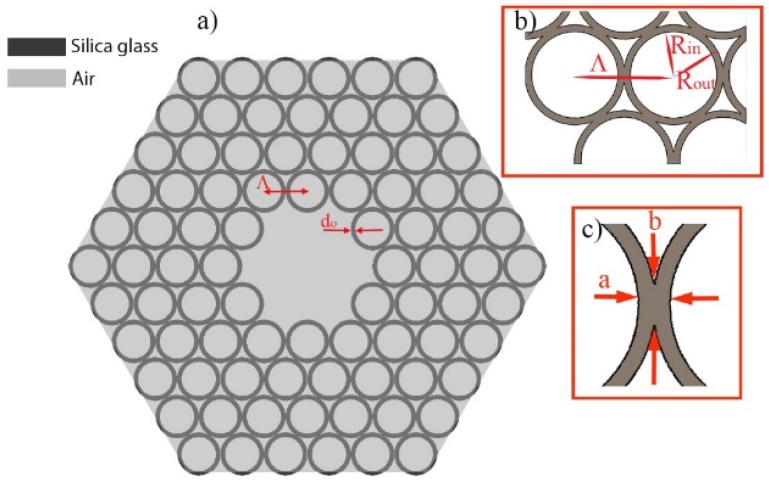
(**a**) Cross section of the designed Kagomé hollow-core fiber, (**b**,**c**) Depicted details of the construction: *R_in_*, *R_out_*, *a*, *b*—inner and outer radiuses of the glass capillaries, and dimensions of the struts, respectively.

**Figure 2:**
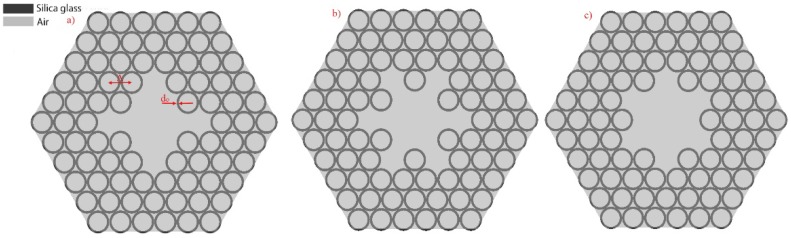
Modified hollow-core fiber structures, based on the 7-cell one (**a**) *xy* structure, (**b**) star structure, and (**c**) a-star structure.

The hexagonal, Kagomé lattice structure that was used for creating the hollow core optical fiber is presented in Figure 1. The fiber’s core is created by removing the seven central, air-filled, silica glass capillaries (cells) of the lattice. The structure’s pitch, *Λ*, is 7.6 μm, while the core radius, *r_core_*, is approximately 9.5 µm. As a material of choice, we used fused silica glass with a refractive index *n_glass_* = 1.454 at 760 nm. By substituting *k* = 1 and *λ* = 760 nm into Equation (1), we calculated the thickness of the capillaries to be *d*_0_ = 540 nm. The choice of *λ* and *k* was dictated by the fact that a wavelength of 760 nm is widely used for multiphoton microscopy and spectroscopy experiments, while by using *k* = 1 we increased the thickness of the capillaries walls, which in turn were easier to fabricate. The inner and outer radius of the capillaries (Figure 1b) were *R_in_* = 3.31 μm and *R_out_* = 3.85 μm, respectively. In this case, the dimensions of the silica struts, resulting from the connection of the adjacent capillaries, are *a* = 0.98 μm and *b* = 1.24 μm, as presented in Figure 1c. The silica struts were treated as a 2D structure with different dimensions, and this approach in our simulations will be further described in Section 3 of this paper. The presented structure is further referred to as ‘7-cell’, and is the basis of the new, modified structures presented in Figure 2. The first structure (Figure 2a), called ‘*xy*’, incorporated the core of the 7-cell structure, enlarged by omitting the additional four cells placed on the x and y axes. The remaining two structures were called ‘star’ and ‘a-star’, and can be seen in Figure 2b,c, respectively. A total of 13 cells were removed in this case—seven from the core, and an additional six cells from the very next cell layer, resulting in an additional space around the core. We will further refer to this space as ‘lateral regions’.

**Figure 3:**
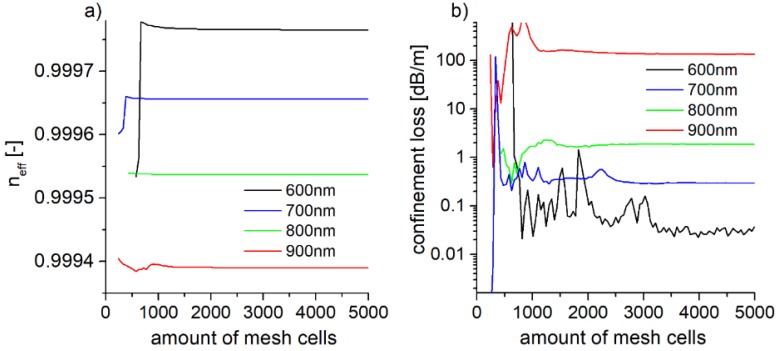
Calculated effective refractive index *n_eff_* (**a**) and confinement loss (**b**) for the fundamental mode of the 7-cell structure, for different amount of mesh cells (*x* direction) and different wavelengths. The appropriate amount of mesh cells in y direction can be calculated from Equation (3). The convergence is obtained if the number of mesh cells is greater than 3000.

The main goal of such a design was to study the influence of removing the glass (or, more generally, reducing the total amount of high-index material) from the vicinity of the core. The pitch and radiuses of the cells for all of the modified structures were the same as in the case of the 7-cell structure. The calculations were conducted by means of the commercially available software Lumerical^®^ MODE Solutions (Lumerical Inc., Vancouver, BC, Canada) [42]. The software uses the finite difference eigenmode (FDE) solver, based on the work of Zhu and Brown [43,44], with rectangular mesh, and the electric and magnetic fields are calculated at each point of the mesh. To provide appropriate simulation accuracy, and to make sure that the periodic structure of the fiber is properly discretized, the total number of mesh cells (in each direction) should be equal to the modeled structure’s *Λ*, multiplied by an integer, and within the span of the simulation region. In this approach, the *x* and *y* spans are both 12 × *Λ*, along the respective axis. However, as increasing the mesh size dramatically increases the calculation time, the convergence tests were conducted to help with the determination of the right size of the mesh (Figure 3). 

By changing the mesh size, calculations of the effective refractive index (*n_eff_*) and *CL*, at four different wavelengths (600–900 nm, with 100 nm step), were conducted. The choice of those particular wavelengths was due to their common use in multiphoton excited fluorescence (MPEF) microscopy and spectroscopy methods, and the fibers were modeled with an intention for future application in both of those fields.

During the simulation, the circular shape of the fiber structure’s glass capillaries should be ensured. Thus, the mesh cells should be square-shaped, having the same dimensions in both axes. Additionally, we have used symmetric and/or anti-symmetric boundary conditions to reduce the simulation time and memory use. The amount of the mesh cells in y direction, *l*, can be calculated from the following equation:

(2) $$l=\mathrm{floor}\left(\frac{y}{x}k\right),$$

where *k* is the amount of the mesh cells in the *x* direction, and *x* and *y* are the sizes of the simulation region in the *x* and *y* directions, respectively.

It can be noticed that if the amount of the mesh cells is bigger than 3100, the *CL* for the chosen wavelengths are stable. For further simulations, we have decided to discretize the simulation region onto a 3192 × 2765 spatial grid. The single cell of the mesh was ~30 nm, while to increase the simulation accuracy without a significant increase of the calculation time, we have imposed an additional mesh in the fiber’s core region (from the structure’s center to the second layer of cladding capillaries), which caused a division of the original, ~30 nm mesh cell by 2, thus reducing the single mesh cell size to ~15 nm. This procedure was inspired by the previously mentioned work of Wang et al. [12], where the dependence of the modal properties of NCHCFs were linked mainly to the geometry of the cladding’s first layer. The simulation parameters (total number of mesh cells in each direction and dimensions of simulation region) were the same for all examined structures. The refractive indices for given wavelengths were obtained using the Sellmeier equations for PMMA [45] and fused silica glass [46].

### 2.2. Fundamental Mode Losses

**Figure 4:**
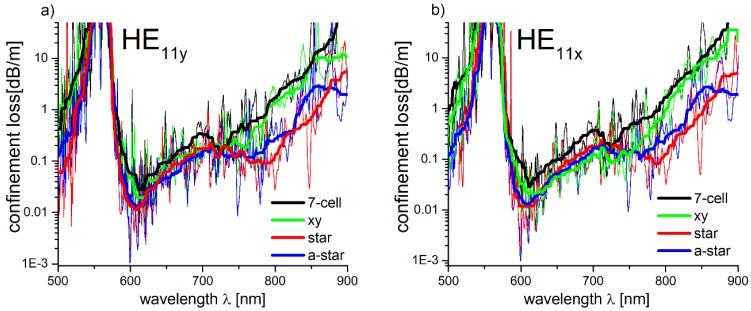
Calculated confinement loss spectra of the fundamental modes (**a**) HE_11x_ and (**b**) HE_11y_ for all simulated structures. In case of the modified structures, i.e., star and a-star, the loss values are clearly reduced for the wavelengths of 750 nm and longer. Additionally, the polarization of the mode (i.e., the *x* or *y* direction) has very little influence on the *CL* spectra of star and a-star structures.

In Figure 4, the *CL* of the fundamental modes HE_11_y (Figure 4a) and HE_11_x (Figure 4b), for each of the simulated structures, are presented. The observed strong oscillations of the *CL* spectra are connected with the coupling between the core and cladding modes. This coupling is mainly dependent on the difference between the cladding and core modes *n_eff_*, as well as their mode field overlaps [32]. To conveniently estimate the relationship between the shape of the core and the *CL* of the modeled fibers, as well as to increase the visual clarity of the presented graphs, the calculated loss spectra were smoothed by means of the percentile filter, with a 40-point window and a percentile equal to 50. It can be noticed that, especially for the star and a-star structures, the *CL* for longer wavelengths (*λ* > 750 nm) are smaller.

The largest reduction of *CL* in the 750–800 nm spectral region, according to the smoothed line, is visible at *λ* = 785 nm for the HE_11y_ mode—~0.08 dB/m and ~0.2 dB/m in case of the star and a-star structures, respectively. Comparatively, the 7-cell and *xy* structures *CL* for the same *λ* are ~0.9 dB/m, which is five to ten times greater than in the case of the modified ones. The results (*CL* spectra of the modeled structures) are similar for the HE_11x_ mode. The *CL* minima for the star and a-star structures occur at *λ* ≈ 790 nm, and their values are ~0.09 dB/m and ~0.2 dB/m, respectively, while the 7-cell and *xy* structures, at the same λ, present nearly identical *CL*—~0.9 dB/m. Based on the results above, the star structure was chosen for further analysis due to its lowest confinement loss.

### 2.3. Dispersion Properties and Effective Mode Area Calculations

**Figure 5:**
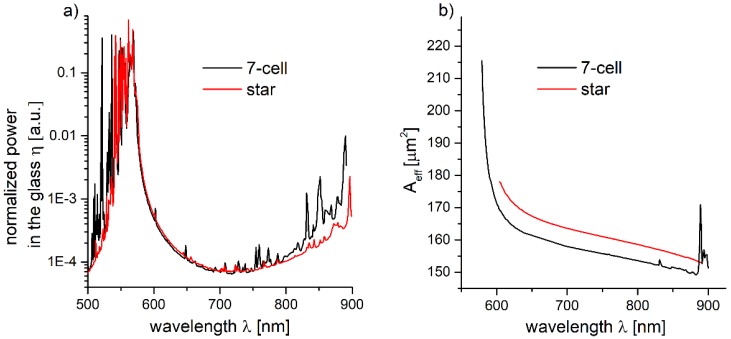
(**a**) Calculated power in the glass (in logarithmic scale), *η*, for the 7-cell and star structures. (**b**) Calculated effective modal areas (*A_eff_*) of the fundamental mode. For both structures, *A_eff_* decreases with the wavelength. The star structure’s *A_eff_* is, in general, slightly bigger in comparison to 7-cell one.

In order to deduce the overlap between the fundamental mode and the glass cladding area, the power in the glass (a fraction of the total power), *η*, can be calculated according to the following equation:

(3) $$\eta =\overset{}{\underset{S_{Si}}{\iint }}p_{z}dS{\left(\overset{}{\underset{S_{\infty }}{\iint }}p_{z}dS\right)}^{-1},$$

where *p_z_* is the longitudinal component of the Poynting vector, while *S_Si_ and S_∞_* indicate integration over the silica glass (or, more generally, the material of the cladding) region and the whole cross section of the modeled structure, respectively. The results of the calculated *η*, as well as the effective mode field area (*A_eff_*) versus the wavelength (*λ*) dependence, are presented in Figure 5a,b, respectively. By comparing Figure 4 and Figure 5a, the correlation between the *CL* and *η* can be noticed.

Especially for longer wavelengths the differences between the star and 7-cell structures, values of *η* are visible. We inferred that the factor that has the strongest influence on the *CL* in this spectral range is the spatial overlap between the core modes and the silica capillaries walls. This overlap is connected with the *A_eff_* of the fundamental mode, which can be defined as [47]:

(4) $$A_{eff}=\frac{{\left(\iint_{-\infty }^{+\infty }{\left|F\left(x,y\right)\right|}^{2}dxdy\right)}^{2}}{\iint_{-\infty }^{+\infty }{\left|F\left(x,y\right)\right|}^{4}dxdy},$$

where *F*(*x*, *y*) is modal distribution of the fundamental mode of the fiber. As one can see in Figure 5b, *A_eff_* is inversely proportional to the wavelength, and this observation was confirmed recently for HC-ARFs with a single-layer-cladding structure [48]. The values of *A_eff_* were used to calculate the dispersion parameter of the fiber. For weakly guiding, radially symmetric optical waveguides, the relation between the spectral dependence of the waveguide dispersion, *β_ω_*_2_, and the mode field radius, *ω_II_*, was originally derived by Petermann [49]:

(5) $$\beta_{\omega 2}=-\frac{\lambda^{3}}{4\pi^{3}c^{2}}\frac{d}{d\lambda }\left(\frac{\lambda }{n_{mat}\omega_{II}^{2}}\right),$$

where *n_mat_* is the material’s index of refraction and *c* is the speed of light in vacuum. This equation was also used for microstructured fibers [50], and assuming the Gaussian shape of the field distribution of the fundamental mode, *A_eff_* = *πω*_0_^2^, Equation (5) yields:

(6) $$\beta_{\omega 2}=\frac{\lambda^{3}}{4\pi^{2}c^{2}n_{mat}}\left(\frac{\lambda }{A_{eff}^{2}}\frac{dA_{eff}}{d\lambda }-\frac{1}{A_{eff}}\right).$$

For hollow core fibers, it can be assumed that the waveguide dispersion is the total dispersion of the fiber, and *D* can be calculated from the following equation [51]:

(7) $$D=-\frac{2\pi c}{\lambda^{2}}\beta_{2},$$

**Figure 6:**
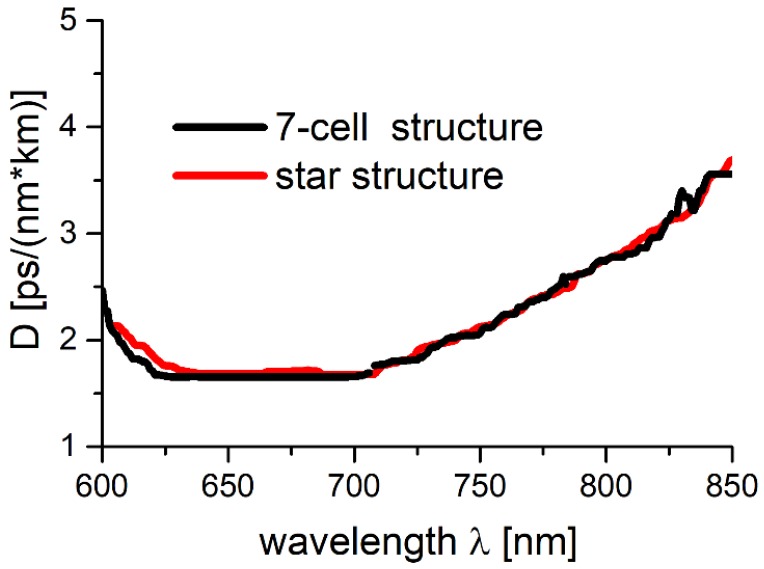
Calculated dispersion parameters *D* for 7-cell and star structures, using Equations (6) and (7). The curves are almost identical in the 600–800 nm range.

In Figure 6, the dependence of *D(λ*), calculated from Equations (6) and (7), for 7-cell and star structures, is presented. One can observe a good agreement between the *D* values for both the 7-cell and star structures, with *D* ranging from 1.5 to 3 ps/(nm × km) in the 700–800 nm region, which is as low as in the case of some previously presented for single-cladding-layer NCHCFs [20,22,30]. These results suggest that both the modeled fibers should be suitable for the purpose of ultrashort laser pulse delivery.

## 3. The Effect of Struts Dimensions

As it was mentioned before, the thickness of the walls of the glass capillaries is a critical factor for the HC-ARFs confinement loss, and it can be calculated using the Equation (1). However, the high-index material layer is not defined only by the walls of these capillaries. During the fiber fabrication process, due to the high temperature, viscosity and gas-pressure (necessary to prevent the collapse of the structure), the walls of the neighboring capillaries form a strut at their contact point [6,38]. These struts have their own dimensions (Figure 1c), which often do not fulfill Equation (1), resulting in the appearance of additional resonant wavelengths, and increasing the fiber’s *CL* and reducing its transmission bandwidth [52]. The size variations of the struts, which may occur during the fiber drawing, can greatly influence the overall loss value of the fiber [53].

**Figure 7:**
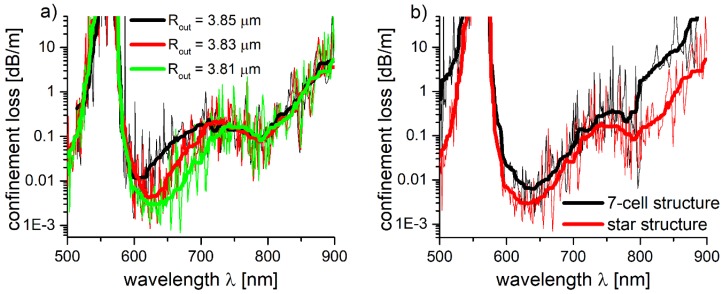
(**a**) Confinement loss of the fundamental modes for star structure, for different values of *R_out_*. (**b**) Confinement loss of the fundamental modes for 7-cell and star structure for *R_out_* = 3.81 µm.

**Figure 8:**
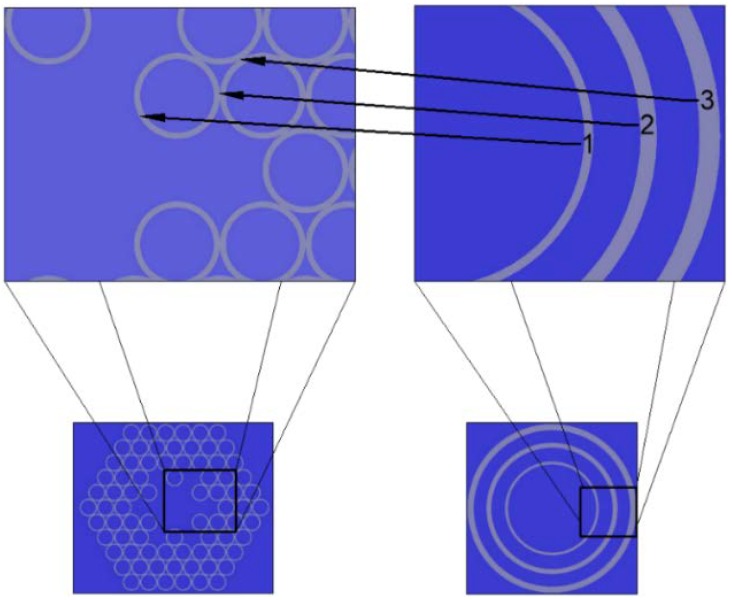
Star structure (**bottom left**) approximated by a triple ring structure (**bottom right**). Each of the glass rings has its wall thickness corresponding to the thickness of the glass element of the star structure, as pointed by the arrows. Consequently, 1 = *d*_0_ = 0.54 µm, 2 = *a* = 0.98 µm and 3 = *b* = 1.24 µm, which corresponds to the outer radius (*R_out_*) of a single glass capillary in the star structure being 3.85 µm.

Figure 7a shows the *CL* spectra of the HE_11_ core mode for star structure, with different values of *R_out_*, while the rest of the parameters were kept unchanged (*Λ* = 7.6 μm, *d*_0_ = 540 nm). On the basis of Figure 1, it is clear that the change of *R_out_* will also cause the change of the dimensions of the struts. The strut dimensions influence is confirmed by Figure 8a results, where one can observe nearly an order of magnitude *CL* reduction in the 600–750 nm wavelength region for different values of *R_out_*. The largest reduction of *CL* was obtained for *R_out_* = 3.81 µm, and thus, the 7-cell and star structures had their initial *R_out_* values changed, and their *CL* spectra were calculated again, as presented in Figure 7b. As expected, the *CL* were reduced for both the 7-cell and star structures, with the first one being influenced significantly more—for example, its lowest *CL* value, in the spectral region of 760–800 nm, is 0.23 dB/m—nearly four times lower than in the case of *R_out_* = 3.85 µm. This effect (in the same spectral region) is not that significant for the star structure—lowest *CL* is 0.08 dB/m, (*λ* = 789nm), compared to 0.09 dB/m for *R_out_* = 3.85 µm, for the same *λ*. 

It is known from the ARROW model that resonances between the cladding layer and the guided light occur at wavelengths, which fulfill the following equation [9]:

(8) $$\lambda_{m}=\frac{2d_{i}}{m}\sqrt{n^{2}-1},$$

where *d_i_* is the thickness of the -*i^th^* cladding layer, *λ_m_* is the -*m^th^* order resonant wavelength (*m* = 0, 1, 2, …), and *n* is the refractive index of the cladding material. The *λ_m_*, calculated for *m* ∊ [0, 5], *n = n_glass_ =* 1.454 and different *R_out_* (corresponding to the change in the strut dimensions), are presented in Table 1. Because *d*_0_ = 540 nm was common for all the structures, its consecutive *λ_m_* values are presented in the last row of the table, without any value of *R_out_* being assigned to it.

Comparing Figure 7a and values in Table 1, one can see the correlation between resonant wavelengths and losses. A large *CL* peak was noticed for all structures at *λ* = 570 nm, where resonance with the first layer of glass capillaries occurred, resulting in strong coupling between the core and cladding modes. For the structure made of capillaries with *R_out_* = 3.85 µm, one can observe additional, weaker resonances for the 650 nm and 680 nm wavelengths, affiliated with the resonances of the struts. The best results were obtained for *R_out_* = 3.81 µm. Although we can see a total of three strut resonances, the ones at *m* = 2 and *m* = 4 effectively contribute to the first-layer one (i.e., then one occurring for *d*_0_ at *m* = 2) which was unavoidable in any way, so no real contribution to the *CL* spectrum was observed. Only the resonance for *m* = 3, occurring at 745 nm, is visible; however, its intensity is still lower when compared with its counterparts, occurring for *R_out_* = 3.83 and *R_out_* = 3.85 µm. It is worth noting that, in this paper, the struts were treated somewhat differently than in the other works, where they are usually considered as a uniform structure with a single dimension [52,53].

As previously mentioned, we have been considering struts as 2D structures, due to fact of their changing orientation, i.e., they rotate (in the geometrical sense) with the structure itself, and in turn the electromagnetic (EM) field ‘coincides’ with their (struts) different dimensions. To ensure the correctness of our interpretation, we have approximated the star structure with the multilayered model of the HC-ARFs, presented recently by Wang and Ding [54]. The structure’s consecutive elements (glass capillary wall, strut dimension *a* and strut dimension *b*, as seen from the center of the structure) have been treated as separate glass layers, each forming a glass ring with a wall thickness equal to the dimension of the given element, and placed in the order mimicking the one from the star structure, as presented in Figure 8.

**Figure 9:**
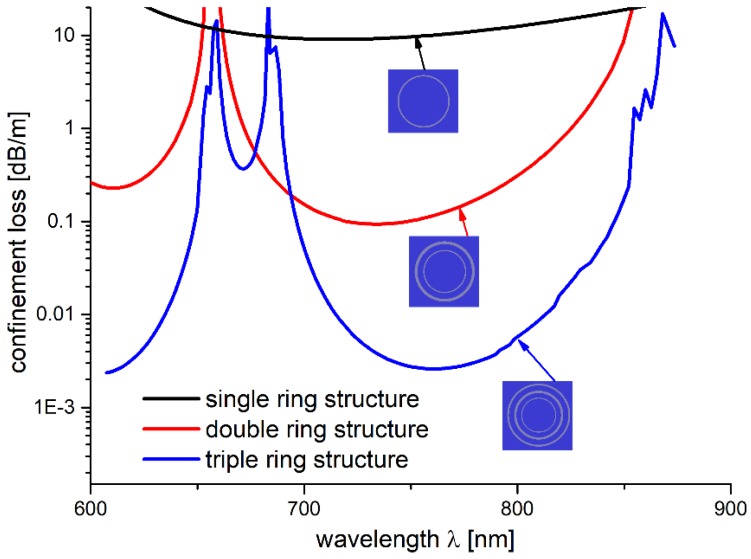
Confinement loss of the single- (black line), double- (red line), and triple- (blue line) ring structure. The innermost ring represents the capillary walls of the star structure, while rings 2 and 3 are a representation of the star structure’s strut. To reflect the strut’s different dimensions, rings 2 and 3 have their thicknesses set to 0.98 and 1.24 µm, which corresponds to the strut’s width (dimension *a*) and height (dimension *b*), respectively. The appearance of three confinement loss peaks (for the triple ring structure), at the resonant wavelengths of ~650, ~690, and ~860 nm, is visible. This peaks correspond to the previously modeled *CL* of the star structure.

Consequently, we calculated the confinement loss for the glass-ring structures with a single, double, and triple glass ring (Figure 9). The *CL* peaks were in good agreement with the ones presented in Table 1 for *R_out_* = 3.85 µm. One can observe two peaks at 650 nm and 680 nm, which correspond to the dimensions *a* and *b* of the star structure struts. The values of the *CL* peaks reflect the order in which the consecutive glass rings appear, resulting in a higher *CL* value for 680 nm. A very similar effect was observed for the star structure in Figure 7a, at *R_out_* = 3.85 μm, with a large increase in *CL* starting at ~610 nm and spanning to ~720 nm. In our opinion, this effect could be explained by the 650 and 680 nm peaks merging and forming a single one, which could be caused by the non-uniformity (in terms of the glass layer thickness) of the star structure, especially when compared to the triple ring one. Nevertheless, the presented results confirmed our initial assumptions about the strut geometry influence on the confinement loss of the simulated structures, and justified treating the struts as 2D objects with different dimensions.

## 4. Bending Losses

**Figure 10:**
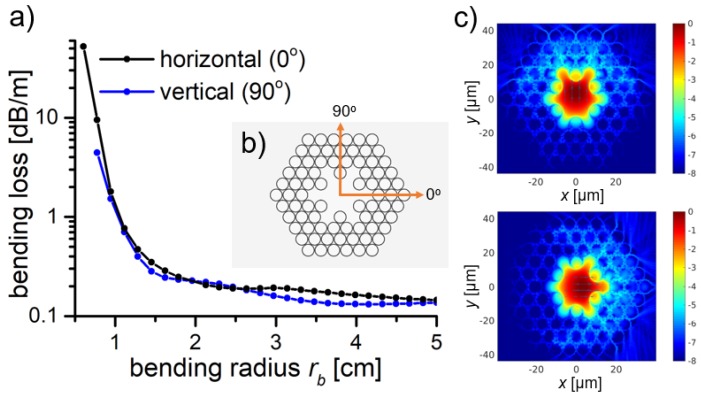
(**a**) Calculated bending losses of the fundamental modes for star structures for different bend orientation; (**b**) Bend-orientation scheme. The orange arrows indicate the direction of the—0° is horizontal, while 90° is vertical bend. (**c**) Modal field distribution, calculated for the fundamental mode at bending radius *r_b_* = 1.3 cm, for vertical (up) and horizontal (down) bend orientation. The color scale bar on the left defines the electromagnetic field intensity. The main reason of the higher bending losses is the coupling between the core and cladding modes, occurring in the direction of the bend.

The bending losses are of great importance for optical fiber sensing applications, such as endoscopy, where small volume cavities are investigated. Thus, in this section, the bending losses for the star structure with *R_out_* = 3.85 µm were calculated for vertical and horizontal bend orientation, at 760 nm wavelength. The obtained results are presented in Figure 10. The *BL* of <0.25 dB/m at a bending radius *r_b_* > 1.5 cm were observed, showing that the designed structure is very bend-resistant.

This result is good enough for the investigation of highly curved, low volume cavities, i.e., for colonoscopy, where the sigmoid colon has about 2.45 cm radius of curvature. Additionally, in Figure 10c, the mode field distribution for the bending conditions of *r_b_* = 1.3 cm, for both bending orientations, is presented. As expected, the direction of bending is mirrored by the increased coupling of the core and cladding modes in the same direction. 

## 5. Anti-Resonant Hollow Core Fibers—PMMA Simulations

**Figure 11:**
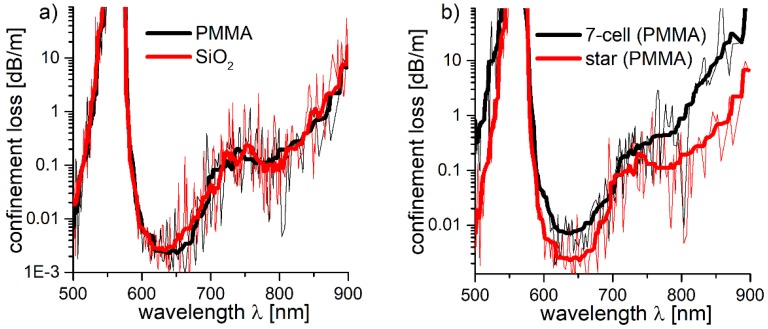
(**a**) Comparison of the confinement loss (*CL*) spectrum of the star structure, based on polymethylmethacrylate (PMMA, black line) and SiO_2_ (silica glass, red line). The spectra are almost identical. (**b**) The *CL* spectrum for the PMMA-based 7-cell and star structures (black and red line, respectively). As in the case of their SiO_2_-based counterparts, a significant reduction of *CL* in the 750–800 nm spectral region is observed for the star structure.

Both the star and 7-cell structures were additionally simulated on PMMA. The amount of mesh cells and *Λ* of the Kagomé lattice remained the same as in previous calculations (3192 × 2765 and 7.6 µm, respectively). The *R_out_* of the lattice capillaries was 3.81 µm, as determined by the results presented in Section 2.3. The capillary wall thickness, calculated from Equation (1), was 520 nm. The *CL* spectra of the SiO_2_ and PMMA-based star structures are almost identical (Figure 11a), with the *CL* minimum of the latter being slightly blue shifted—from 790 nm to ~780 nm. PMMA-based star and 7-cell structures were also compared (Figure 11b), and the star structure exhibited *CL* reduction from ~0.5 dB/m to ~0.1 dB/m (*λ* = 780 nm), similarly to SiO_2_.

**Figure 12:**
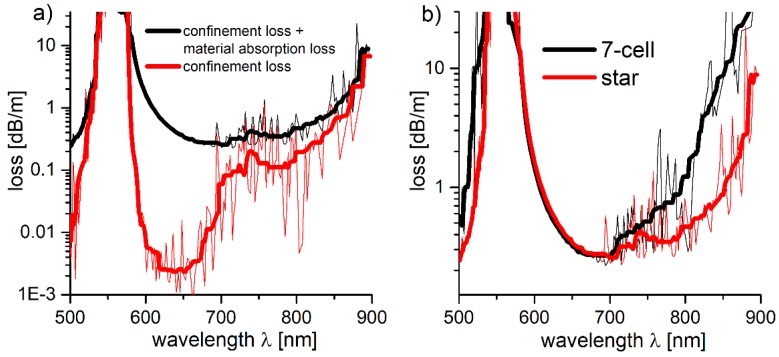
(**a**) Comparison of the loss (confinement loss + material absorption loss, black line) and confinement loss spectra (red line) of the star structure based on PMMA. The loss increase is visible, particularly in the 600–700 nm region, which suggests that the total loss here is influenced mainly by the PMMA material absorption. (**b**) Loss spectra of the PMMA-based 7-cell and star structures (black line and red line, respectively). In the 600–700 nm spectral region the influence of the material absorption loss is large enough to negate the confinement loss decrease of the star structure. As the wavelength increases, the latter again starts to outperform the 7-cell one.

As the material absorption of PMMA in the UV-NIR region [32] can play a significant role in the fiber’s total loss value, additional loss simulations considering the PMMA’s refractive index’s imaginary part (extinction coefficient, *k*), which is directly responsible for the materials absorption, were conducted. Using the Lumerical’s^®^ (n,k) Material Model [55], and assuming constant *k* for the given wavelength range (*k* = 3 × 10^−5^ for *λ* = 600–850 nm, retrieved from [56]), total losses of the PMMA star structure were calculated. The obtained results, compared with the previously modeled *CL* from Figure 11, are presented in Figure 12a. The total increase in the loss is visible, especially in the 600–700 nm wavelength region. However, the value of *k* used for the simulations was relatively high when compared with the values obtained by other researchers, i.e., [57], where *k* is 2 orders of magnitude lower.

Nevertheless, these results still show that this structure has a potential to transmit the NIR light, even if made from PMMA. The obtained loss values in the spectral region of 750–800 nm (0.37 dB/m and 0.43 dB/m, respectively) are significantly lower than in the case of the solid-core POFs (~0.6 dB/m at 750 nm and ~2 dB/m at 800 nm, according to [58]). In Figure 12b, where the total loss of 7-cell and star structures are compared, one can observe that the previously shown (Figure 11b) *CL* reduction is not that significant in the spectral region of 600–700 nm, which suggests that the material absorption losses are of major relevance there. However, as the wavelength becomes longer, the star structure starts to regain advantage over the 7-cell one, as its losses are significantly lower for the 700–850 nm wavelength range (~1 dB/m at *λ* = 850 nm, compared to ~10 dB/m at the same *λ* in the case of 7-cell structure).

**Figure 13:**
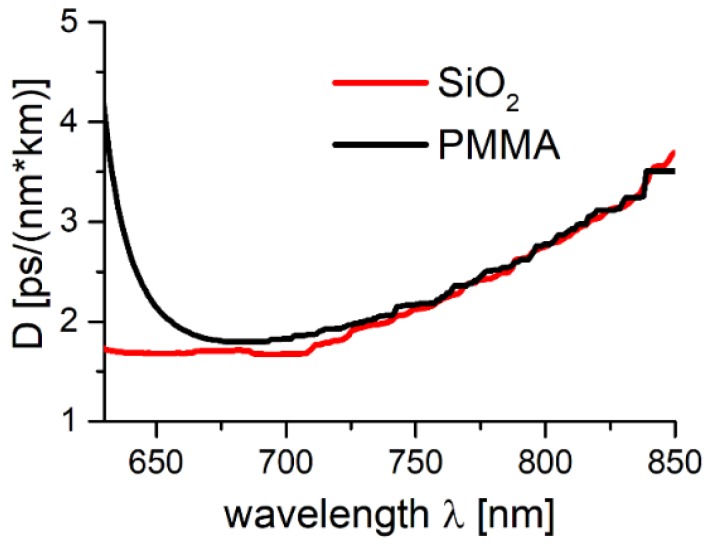
Dispersion parameter spectrum for the star structure made of PMMA and silica glass (SiO_2_). For the spectral range of ~675 to 800 nm, both dispersion curves are almost identical. As a result, both fibers can be considered suitable for ultrashort light pulse delivery.

Solid core POF’s can have their material dispersion as high as few hundred ps/(nm × km) [59], and thus are unsuitable for ultrashort laser pulse delivery. As the values of *D* presented in Section 2.3 suggest that the modeled structures can overcome the problem of material dispersion, we recalculated the *D*(*λ*) dependence, switching the refractive index of fused silica with the one of PMMA. As a result, we obtained the values of the PMMA-based star structure’s dispersion parameter (*D_PMMA_*(*λ*)), and compared them with the results from Figure 6. This comparison is presented in Figure 13, and clearly shows that the differences between both structures, in terms of their *D* values, are almost negligible in the spectral window of 700–800 nm, suggesting that the star structure could indeed reduce the problem of PMMA material dispersion, and open the field of dispersion-free, ultrashort optical pulse delivery though mPOFs.

## 6. Conclusions

In this paper, we reported a numerical study of several structures of hollow core fibers with a modified shape of the core-surrounding capillaries region. The cores of these fibers are formed by removing some of the silica capillaries located in the direct vicinity of the core. Introducing such distortions to the structure allows a reduction of the confinement losses of the fundamental modes due to the smaller overlap between core modes and the cladding material area. We also showed how the different dimensions of the structure’s struts influence the fiber’s *CL* spectrum. One sees that struts introduce additional resonant frequencies, which manifest themselves by increasing the total fiber losses in their spectral region of appearance. This results were additionally confirmed by using the multi-layered model to approximate the proposed structure. We have also investigated the bending loss of the proposed fibers, which have appeared to be as low as 0.25 dB/m for a bending radius of *r*_0_ = 1.5 cm, which is good enough to consider these fiber structures for applications such as endoscopy or others. Finally, we have presented that, theoretically, such a fiber structure, if made from PMMA, would keep its key optical properties—reduced loss and extremely low dispersion parameter in the visible-near infrared spectral region, effectively making the field of ultrafast optics much more accessible for the mPOFs. 

## Figures and Tables

**Table 1:** Resonant wavelengths (*λ_m_*) calculated for different values of the capillary outer radius (*R_out_*). Values in red are the resonant wavelengths of particular interest to the conducted research, as they directly influence the transmission bandwidth of the modeled hollow-core anti-resonant fiber (HC-ARF) structures, and can be seen in their *CL* spectra in the form of regions of increased *CL*.

*R_out_* [µm]	Strut Dimensions [µm]	Resonant Wavelengths *λ_m_* of *m^th^* Order [µm]				
		*m* = 1	*m* = 2	*m* = 3	*m* = 4	*m* = 5
3.85	*a* = 0.98	2.07	1.034	0.68	0.51	0.41
	*b* = 1.24	2.60	1.31	0.87	0.65	0.52
3.83	*a* = 1.02	2.15	1.07	0.71	0.54	0.43
	*b* = 0.96	2.02	1.01	0.67	0.50	0.4
3.81	*a* = 1.06	2.24	1.11	0.745	0.56	0.44
	*b* = 0.55	1.16	0.58	0.38	0.29	0.23
-	*d_0_* = 0.54	1.14	0.57	0.38	0.28	0.23
